# Phenotypic variability and genetic diversity analysis of cultivated potatoes in China

**DOI:** 10.3389/fpls.2022.954162

**Published:** 2022-09-23

**Authors:** Jun Hu, Meng Mei, Fang Jin, Jianfei Xu, Shaoguang Duan, Chunsong Bian, Guangcun Li, Xiyao Wang, Liping Jin

**Affiliations:** ^1^ Key Laboratory of Biology and Genetic Improvement of Tuber and Root Crops, Ministry of Agriculture and Rural Affairs of the People’s Republic of China, Institute of Vegetables and Flowers, Chinese Academy of Agricultural Sciences, Beijing, China; ^2^ College of Agronomy, Sichuan Agricultural University, Chengdu, China; ^3^ National Agro-Tech Extension and Service Center, Ministry of Agriculture and Rural Affairs of the People’s Republic of China, Beijing, China

**Keywords:** potato, SSR marker, genetic diversity, population structure, variety identification

## Abstract

Phenotypic evaluation and molecular biotechnology are both important in the identification and utilization of crop germplasm resources. In this study, the phenotypic variation and genetic diversity of 149 main potato cultivars in China were investigated with 12 phenotypic traits and 24 SSR markers. The coefficient of variation of 12 phenotypic traits ranged from 12.11% to 156.93%. The results of SSR markers exhibited a relatively high level of genetic variation (*Na* =5.458 ± 1.499, *Ne* =3.300 ± 1.087, *I* =1.397 ± 0.298, *Ho* =0.797 ± 0.178, *He* = 0.660 ± 0.117, and *PIC*=0.702 ± 0.087). Population structure and phylogenetic tree analysis divided the varieties into three subgroups. The results indicated that ninety percent of the molecular variance was attributed to within-group differences, and the remaining 10% was attributed to variation among groups. Consistent with previous report, alleles of the STI032 marker were significantly associated with tuber starch content and growth period traits in the population. The results of this study could facilitate the utilization of potato germplasm resources, molecular genetic breeding and improvement.

## Introduction

China produces 25.5% of the world’s potato (FAOSTAT, 2020). With a total acreage of 4.2 million hectares and production of 78.2 million tons in 2020 (FAOSTAT, 2020), potatoes are widely distributed throughout the country in four agro-ecological zones, including the Northern single-cropping zone, the Southwestern mixed-cropping zone, the Central Plain double-cropping zone and the Southern winter-cropping zone (Jansky et al., 2009). There are different potato breeding objectives for key traits of varieties in different zones (Jansky et al., 2009). For example, early maturity and yellow tuber flesh are favored in the Central Plain double-cropping zone and the Southern winter-cropping zone, whereas late maturity and high tuber starch contents are common in the Northern single-cropping zone. In addition, high resistance to late blight is necessary in the Southwestern mixed-cropping zone (Jansky et al., 2009). Since the 1960s, more than 800 new potato varieties have been released from breeding programs in China, most of which are suitable for table consumption (Xu and Jin, 2017). It is still difficult to meet the demands for the development of the potato processing industry in China.

The effective exploitation of plant genetic resources is built on the accurate identification of phenotypic and genotypic variation from target germplasms. Determination of genetic diversity is helpful for the effective use of germplasm, especially in plant breeding research. There have been some studies on potato germplasm and variety at both the morphological and molecular levels (Wang et al., 2017; Duan et al., 2019; Wang et al., 2019; Duan et al., 2021). A total of 288 potato genotypes from around the world were classified into three to eight groups with 20 SSR and 10 AFLP markers (Wang et al., 2017). A population of 292 genotypes from the International Potato Center (CIP), Europe and Yunnan Province in China was clustered into two main groups and subdivided into seven groups using 30 SSR markers (Wang et al., 2019). However, the systematic phenotypic variability and genetic diversity and population structure of potato varieties bred from various regions of China need more study.

Pedigree analysis is an important method for selecting parents and confirming the genetic relationships of their offspring (Li et al., 2018a). However, due to the lack of effective pedigree information, it is difficult to determine genetic relationships by the pedigree method in many varieties (Duan et al., 2019). Molecular genetic diversity studies can evaluate all levels of genetic structure from the relationship between complex components of species to the origin of specific genotypes. Different marker systems, such as SSRs, AFLPs and SNPs (single nucleotide polymorphisms), have been employed to study different crops in addition to potato. However, traditional SSR marker polymorphisms are analyzed by polyacrylamide gel electrophoresis (PAGE) and manual reading, which is time-consuming, labor-intensive and nonautomated. Moreover, data collection and analysis of multiple batch samples are associated with numerous difficulties. Capillary electrophoresis with fluorescent-labeled SSR primers has been widely used (Chandra et al., 2014; Liang et al., 2018; Tiwari et al., 2018; Verma et al., 2019; Ayenan et al., 2021) owing to its high efficiency and automation.

Plant maturity is an important agronomic trait in potato. Maturity is related to starch content and late blight resistance, which is an important factor in determining the regional adaptability of varieties (Domański, 2001; van Eck, 2007). Studies focused on quantitative trait loci (QTLs) for plant maturity both in diploid and tetraploid potato have been reported (Bradshaw et al., 2008; McCord et al., 2011; Kloosterman et al., 2013; D’Hoop et al., 2014; Massa et al., 2015; Li et al., 2018b). A major QTL related to potato plant maturity was located on chromosome V (Kloosterman et al., 2013; Li et al., 2018b). Two overlapping QTLs for potato maturity and tuber dry matter content were identified on chromosome V in a tetraploid population (Bradshaw et al., 2008). Fourteen loci for tuber starch content were identified on seven chromosomes by association analysis in 243 tetraploid potato germplasms (Li et al., 2008). Although an abundance of excellent genetic resources exist in diploid wild germplasm (Duan et al., 2021), it is difficult to deploy these resources in tetraploid cultivated varieties by introgression due to reproductive barriers and/or unfavorable linkage drag.

In this study, we examined the phenotypic variability and genetic diversity of 149 main potato cultivars to uncover their genetic diversity and relationships among populations for the utilization and identification of potato germplasm and breeding programs. The information in the study provides data support for variety identification and selection of cross-combinations among cultivars to improve potato breeding in China.

## Materials and methods

### Plant materials and phenotypic traits

A brief description of the 149 main potato varieties grown in China since the 1970s is summarized in **Supplementary Table S1**. Of the 149 cultivars used in the study, 144 were developed by breeding programs in different regions of China, and five (Atlantic, Favorita, Shepody, Mira and Desiree) were imported from abroad and commonly planted in China. The phenotypic data of the varieties were collected from the information provided by the national variety approval and registration in China through a big data platform (http://202.127.42.47:6010/index.aspx) and the National Potato Variety Resources Catalogue (Hongzhu Yang, 1989). Growth period (GP), plant height (PH), tuber dry matter content (DM), starch content (SC), reducing sugar content (RS), protein content (PC) and vitamin C content (VC) data were recorded as numerical values in the database. Tuber shape (TS), eye depth (ED), tuber skin color (TSC), tuber flesh color (TFC) and flower color (FC) were determined according to Domański L. (2001) with minor modifications. TS was scored as follows: 1: compressed, 2:  round, 3: round-oval, 4: oval, 5:  long-oval, and 6:  long. ED was evaluated on a scale ranging from 1 to 9, where 1: greater deep bud eye, 3: deep bud eye, 5: moderate bud eye, 7: shallow bud eye and 9: greater shallow bud eye. TSC and TFC were evaluated according to a 0- to 5-point scale, where 0: white, 1: pale yellow, 2: yellow, 3: deep yellow, 4: red, and 5: purple. FC was scored as follows: 0: white, 1: pale violet, 2: violet, 3: dark violet, and 4: blue corolla. Analysis of word cloud was finished by *wordcloud* package in R software to determine the frequency of a potato cultivar used as a parent. Network analysis was performed using the *igraph* package in R software.

### DNA extraction and SSR analysis

Approximately 2 g of fresh young leaves was collected. Genomic DNA was extracted according to the modified cetyltrimethylammonium bromide (CTAB) procedure. All DNA samples were diluted to 25 ng μL^-1^ and stored at -20°C until use. Twenty-four SSR primer pairs covering all 12 chromosomes were labeled with FAM, HEX, ROX and TAMRA fluorescent dyes reseparately. Primers were synthesized by Qingke Biotech Company (Beijing, China). The sequences of these primer pairs were obtained from previous reports (**Supplementary Table S2**). PCR amplifications were performed in a 20 μL reaction mixture that consisted of 4.0 μL of DNA template, 0.5 μL of forward primer (10 pmol μL^-1^), 0.5 μL of reverse primer (10 pmol μL^-1^), 10 μL of 2 × Taq Master Mix (Vazyme, Nanjing, China), and 5 μL of ddH_2_O. A touchdown PCR was used: 5 min at 95°C, 13 cycles of 30 s for denaturation at 95°C, 45 s for gradient annealing from 60°C to 50°C (each cycle reduced by 0.8°C), 30 s for extension at 72°C, followed by 24 cycles of 95°C for 30 s, 50°C for 45 s, and 72°C for 30 s, with a final step at 72°C for 5 min. The amplified SSR products were fragment analyzed on a ‘3730 Genetic Analyzer’ (Applied Biosystems, Foster City, California, USA). The results of the peak patterns produced were analyzed by an SSR Analyzer (Wang et al., 2018), which is based on the commercialized software GeneMarker. A 500-bp LIZ500 standard was used to estimate the molecular size of the amplification fragments. For each locus, peaks were recorded in order from smallest to largest (**Supplementary Table S3**). The number of peaks and the number of profiles per marker were scored based on amplification of the cultivars. A data matrix of 149 cultivars was constructed based on the presence (1) or absence (0) of the amplified SSR fragments.

### Genetic diversity, population structure and phylogeny analysis

The number of alleles (*Na*), the effective number of alleles (*Ne*), Shannon’s information index (*I*), observed heterozygosity (*Ho*), expected heterozygosity (*He*), and *F*-Statistics (*Fis*) were determined using the GenAlEx 6.502 program (Peakall and Smouse, 2012), whereas the polymorphism information content (*PIC*) value was estimated using PowerMarkerv3.25 software (Liu and Muse, 2005). Population structure was inferred by Bayesian clustering implemented in STRUCTURE v.2.3.4 (Pritchard et al., 2000). K values from 1 to 20 were tested for twenty independent runs with a burn-in length of 100,000 and MCMC repetitions of 100,000. The most likely K value was analyzed by the method in Evanno et al. (2005) using STRUCTURE Harvester (Earl and Vonholdt, 2012). Furthermore, analysis of molecular variance (AMOVA) was conducted to estimate variation among the three groups using GeneAlEx software with 1000 permutations. A cluster analysis based on the neighbor-joining method was also conducted using PowerMarker software, and then an unrooted tree was constructed and analyzed using MEGA X software with default setting parameters (Kumar et al., 2018).

### Marker−trait associations

Association analysis was carried out for the identification of significant marker−trait association (MTA). The phenotype trait data along with SSR markers were analyzed in the TASSEL 5.2.77 program (Bradbury et al., 2007) to identify significant marker−trait associations (MTAs). The analysis of MTAs was performed using a mixed linear model (MLM) with TASSEL software based on the Q-matrix and kinship matrix (K-matrix). The relative kinship matrix (K) was determined using SPAGeDi software v1.2 (Hardy and Vekemans, 2002). The significance of MTAs was tested in terms of *P* value (*P* < 0.01 for significant markers). False discovery rate (FDR) was analyzed using the Q-values in R 3.5.3 (R Core Team, 2019).

## Results

### Analysis of phenotypic traits and pedigree information

**Figure 1:**
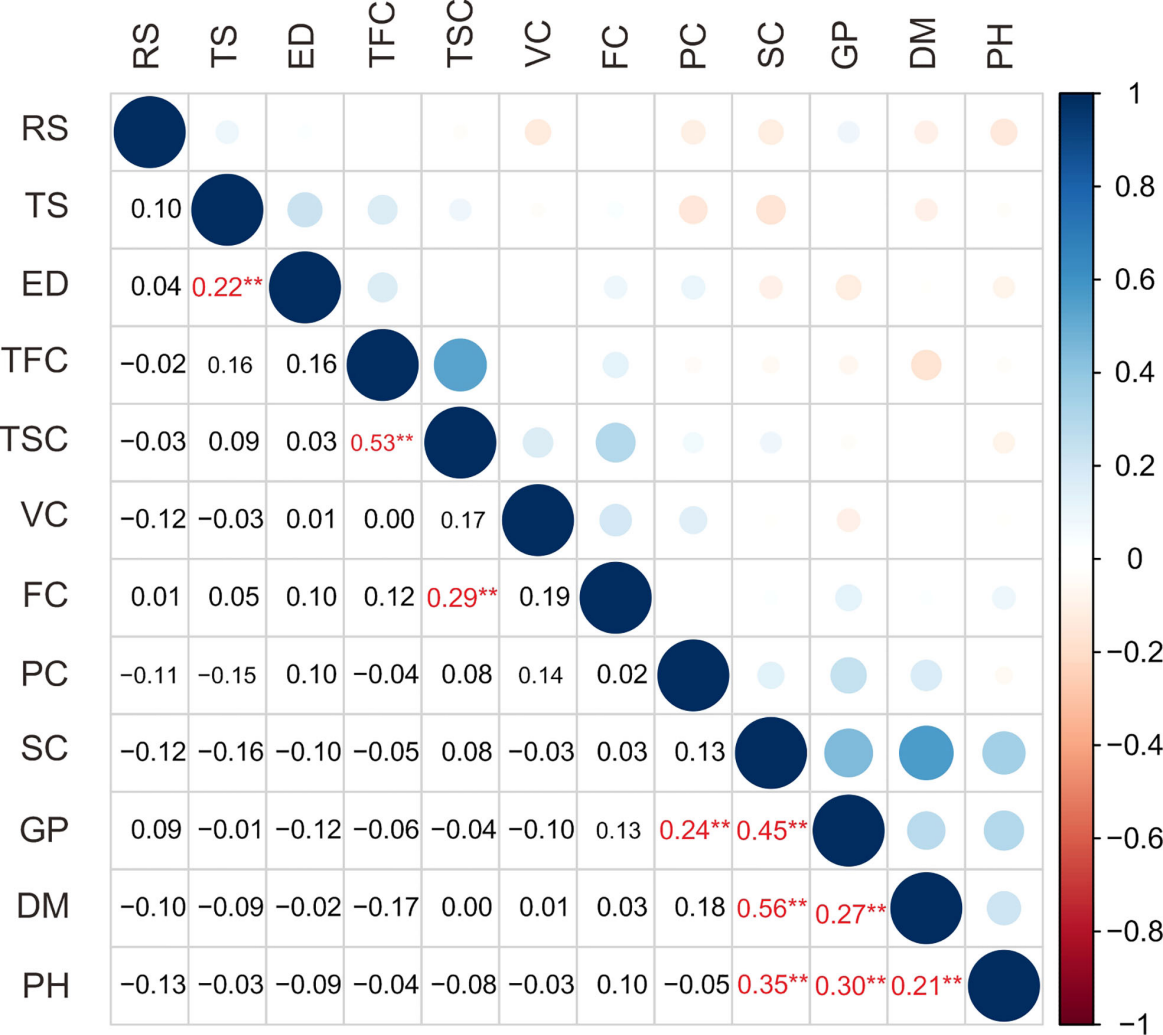
Correlation analysis of 12 traits of 149 potato cultivars. RS, reducing sugar content; TS, tuber shape; ED, eye depth; TFC, tuber flesh color; TSC, tuber skin color; VC, vitamin C content; FC, flower color; PC, protein content; SC, starch content; GP, growth period; DM, dry matter content; PH, plant height. The numbers shown in red reach extremely significant correlation and are indicated with ** (P<0.01).

In this study, a total of 12 traits, such as growth period (GP), plant height (PH), flower color (FC), tuber shape (TS), tuber skin color (TSC), tuber flesh color (TFC), eye depth (ED), starch content (SC), dry matter content (DM), protein content (PC), reducing sugar content (RS) and vitamin C content (VC), in the population are listed in **Table 1**. The coefficient of variance for the traits ranged from 12.11% in dry matter content to 156.93% in flower color (**Table 1**). The analysis of the traits revealed a broad spectrum of variation in the population. Correlation analysis showed 10 extremely significant (*P* < 0.01) differences in correlation between traits (**Figure 1**, **Supplementary Table S4**). Growth period traits showed an extremely significant correlation with the tuber dry matter content and starch content (*P* < 0.001) (**Figure 1**). Principal component analysis of the phenotypic data of the 12 traits showed that six principal components potentially contributed to the variation of the traits, with a cumulative variance of 71.3%. The eigenvalue of the first principal component was 1.504, accounting for 18.97% of the total variation, and the eigenvalue of the second component was 1.333, accounting for 14.92% of the total variation in the population (**Supplementary Table S5**). Three main groups were detected on the cluster dendrogram in accordance with phenotypic trait values. From up (above) to down (below), the first cluster contained 7 cultivars, the second 76 cultivars, and the third 66 cultivars (**Supplementary Figure S1**).

**Table 1:** 12 phenotypic variation parameters of 149 potato cultivars in China.

Traits	Min.	Max.	Average	SD	CV
Growth Period (d)	50.00	144.00	97.38	19.85	20.39
Plant Height (cm)	32.00	113.00	62.80	13.44	21.40
Starch Content (%)	10.30	22.86	15.49	2.49	16.06
Dry matter Content (%)	14.80	28.20	21.76	2.64	12.11
Reducing Sugar Content (%)	0.02	1.54	0.35	0.25	72.57
Vitamin C Content (mg.100 g^-1^)	8.93	48.00	17.50	5.72	32.68
Protein Content (%)	0.53	3.52	2.16	0.44	20.59
Flower Color	0.00	4.00	0.62	0.98	156.93
Tuber Shape	1.00	6.00	2.76	1.46	53.07
Eye Depth	2.00	9.00	6.36	1.26	19.80
Tuber Skin Color	0.00	5.00	1.60	1.12	69.87
Tuber Flesh Color	0.00	5.00	0.92	0.88	96.40

SD, standard Deviation; CV, Coefficient of Variance.

**Figure 2:**
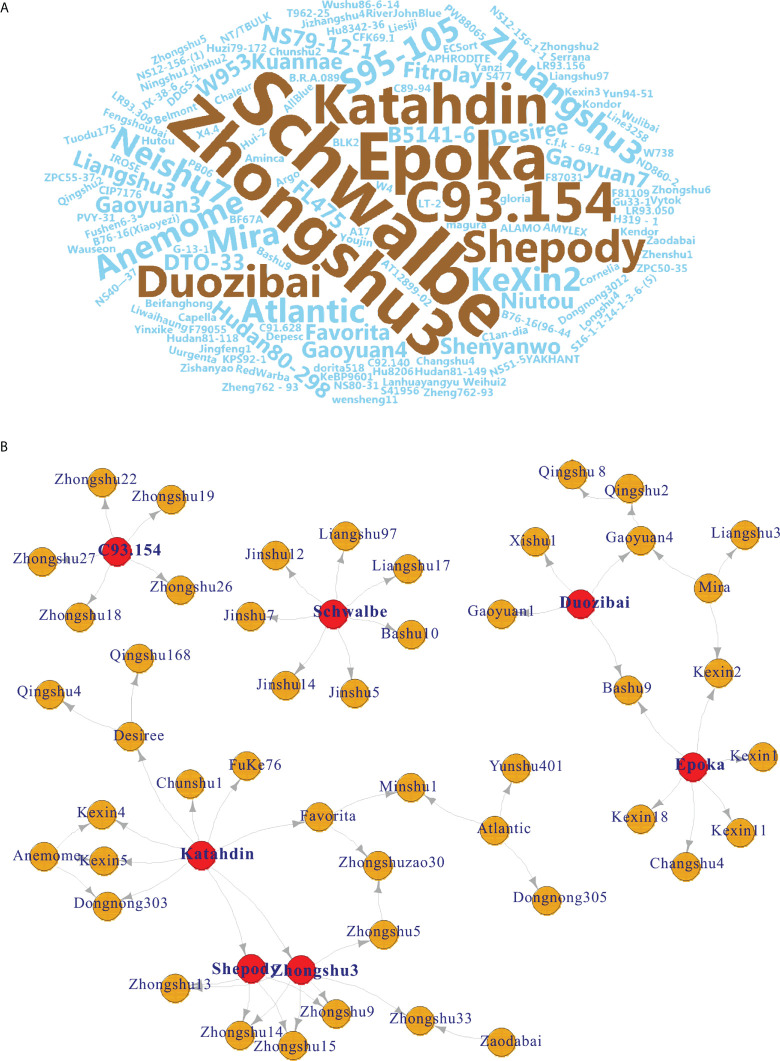
Pedigree-based word cloud and network analysis. **(A)** Pedigree-based word cloud analysis of 149 potato varieties; the most frequently used parents are shown in brown. **(B)** Network of seven high-frequency potato varieties (red node) based on the results of the word cloud.

Seven potato cultivars were frequently used as parents by word cloud analysis (**Figure 2A**). Among them, cv. Schwalbe was used the most frequently used (n = 7 times), followed by the cultivars Zhongshu 3 (n = 6), Epoka (n = 6), C93.154 (n = 5), Katahdin (n = 5), Duozibai (n = 4), and Shepody (n = 4) (**Figure 2B**).

### SSR polymorphism

A total of 148 SSR markers, including 77 markers from previous studies (Kishine et al., 2017; Duan et al., 2019; Wang et al., 2019; Duan et al., 2021) and 71 new trinucleotide and tetranucleotide core motif SSR markers, were used for primer screening with sixteen cultivars based on polyacrylamide gel electrophoresis (data not shown). Finally, twenty-four SSR markers from all 12 chromosomes (**Supplementary Table S2**) were screened out to evaluate the genetic diversity of 149 potato cultivars. A total of 131 alleles were recorded. The number of alleles per locus (*Na*) varied from 2 to 9, with a mean of 5.46 ± 1.50. The effective number of alleles per locus (*Ne*) ranged from 1.64 to 5.29, with a mean of 3.30 ± 1.09. The value of observed heterozygosity (*Ho*) changed from 0.46 to 0.994 with an average value of 0.797, while the value of expected heterozygosity (*He*) varied from 0.392 to 0.811 with a mean value of 0.66. The mean of polymorphism information content (*PIC*) was 0.702 ± 0.087, and Shannon’s information index (*I*) varied from 0.733 to 1.76 (mean 1.397 ± 0.298). The value of the inbreeding coefficient (*F_IS_*) ranged from − 0.359 to 0.551 (mean -0.026 ± 0.253) (**Table 2**).

**Table 2:** Diversity information parameters of 149 cultivated potato genotypes using 24 SSR markers.

Marker	*Na*	*Ne*	*He*	*Ho*	*I*	*PIC*	*F_IS_*
31924	6	4.45	0.776	0.969	1.596	0.780	-0.226
43016	7	2.57	0.611	0.516	1.441	0.680	0.449
S118	5	3.39	0.705	0.783	1.387	0.715	0.157
S151	6	3.93	0.746	0.913	1.577	0.767	-0.152
S170	6	4.74	0.789	0.988	1.629	0.790	-0.238
S182	5	3.66	0.727	0.907	1.397	0.697	-0.144
S187	5	3.15	0.683	0.888	1.356	0.707	-0.164
S189	9	5.29	0.811	0.994	1.760	0.813	-0.218
S192	4	2.35	0.574	0.733	1.175	0.619	-0.034
S7	7	4.66	0.786	0.925	1.706	0.794	-0.118
SSR08337	4	2.29	0.563	0.702	1.096	0.614	0.070
STG0025	2	1.99	0.498	0.857	1.726	0.654	-0.359
STG0026	4	2.39	0.582	0.795	1.072	0.622	-0.126
STI0012	7	4.25	0.765	0.969	1.641	0.779	-0.223
STI017	5	3.79	0.736	0.988	1.632	0.764	-0.320
STI032	6	4.42	0.774	0.969	1.678	0.790	-0.210
STM0037	6	5.19	0.807	0.988	1.711	0.809	-0.212
STM1049	4	1.79	0.441	0.571	0.786	0.655	0.137
STM1104	6	2.53	0.604	0.658	1.271	0.649	0.252
STM1106	8	2.82	0.645	0.497	1.573	0.713	0.496
STM2022	6	2.99	0.666	0.708	1.447	0.742	0.006
STM3012	5	2.8	0.643	0.857	1.241	0.678	-0.173
STM5121	4	2.11	0.526	0.460	0.908	0.547	0.551
STPoAc58	4	1.64	0.392	0.491	0.733	0.462	0.183
Mean	5.46	3.30	0.660	0.797	1.397	0.702	-0.026

Na, the number of alleles per locus (Na); Ne, the effective number of alleles per locus; Ho, observed heterozygosity; He, expected heterozygosity; I, Shannon’s information index; PIC, polymorphism information content; F_IS_, inbreeding coefficient.

### Population genetic diversity

**Figure 3:**
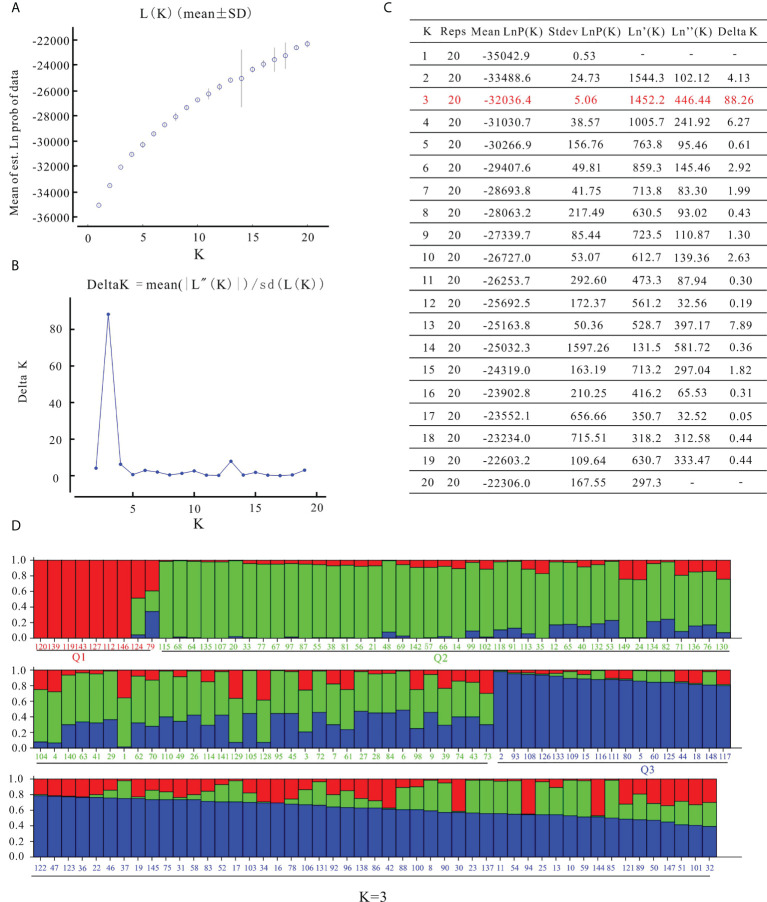
Analysis of population structure of 149 potato varieties. **(A)** Mean log-likelihood [Ln(K) ± SD], **(B)** △K values, **(C)** each K value based on the model reported in the article Evanno et al. (2005), **(D)** population structure of 149 potato varieties on K values of 3. The colored bar grouped the varieties in the corresponding populations, red, Q1 group; green, Q2 group; blue, Q3 group.

**Figure 4:**
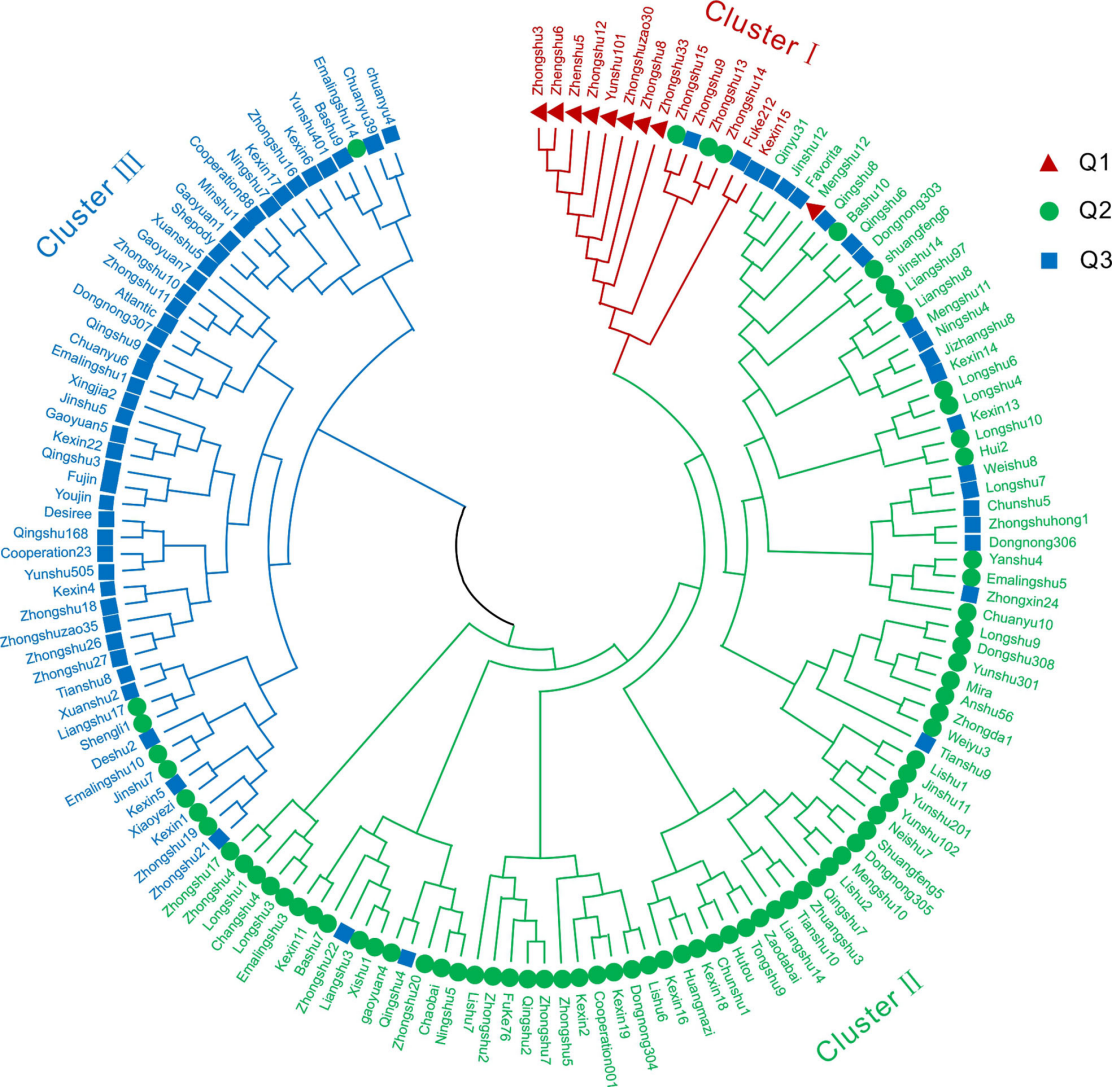
Phylogenetic tree of 149 potato varieties. A phylogenetic tree of all 149 potato varieties analyzed using 24 SSR markers was constructed by the neighbor-joining method. Cluster I included 14 varieties shown in red, cluster II included 85 varieties shown in green, and cluster III contained 50 varieties shown in blue. Q1, Q2 and Q3 represent the three groups of classification results of Structure analysis.

STRUCTURE software was used to characterize the population structure. The log-likelihood value LnP(D) continued to increase without clear inflection as K increased from 1 to 20 (**Figure 3A**). When using the ΔK method (Evanno et al., 2005), the peak value appeared at K = 3 (**Figures 3B, C**). In the results of Struture analysis (**Figure 3D**), there were 9 varieties in the Q1 group, 74 varieties in the Q2 group, and 66 varieties in the Q3 group. According to phylogenetic trees based on the neighbor-joining method, the 149 varieties were classified into three clusters (**Figure 4**). The first group (Cluster I) consisted of 14 varieties, including 8 varieties from the Q1 group, such as ‘Zhongshu 3’, ‘Zhongshu 8, ‘Zhongshu 12’, ‘Zhongshuzao 30’, ‘Zhongshu 33’, ‘Yunshu 101’, ‘Zhengshu 5’ and ‘Zhengshu 6’ (**Figure 4**). Cluster II (green) consisted of 85 genotypes (**Figure 4**), including 63 varieties from the Q2 group. There were 50 varieties in cluster III (blue), including 42 varieties from the Q3 group. The genetic diversity of 149 varieties was also analyzed by PCoA. The results also classified the 149 potato varieties into three major groups (**Supplementary Figure S2**), which agreed with the results of STRUCTURE. The cultivars in Q1 were grouped in the upper-left corner of the plot, and the genotypes in Q2 were distributed in the lower-left part of the plot. The genotypes in the Q3 subgroup were distributed on the right side of the plot. Molecular variance analysis indicated that the major proportion of the variance was due to variation within groups, and the remaining 10% was due to variation among groups (**Supplementary Table S6**).

### Identification of marker−trait associations

Based on the phenotypic traits and genotype data, associations between markers and traits were analyzed through TASSEL software using a mixed linear model (MLM). We performed 1572 (131 SSR alleles × 12 traits) marker-trait association tests. Thirty-three associations (2.09%) reached a significant level (P < 0.01), and the number was reduced to five (0.32%) after the false discovery rate (FDR) method test (Q < 0.05). Three loci (STI032_4, STI032_6 and SSR08337_1) were associated with starch content traits, explaining 7.31% to 12.56% of the variation. Two loci (STI032_4 and STI0012_3) were associated with the growth period, explaining 9.72% to 11.01% of the variation, respectively (**Table 3** and **Supplementary Figure S3**).

**Table 3:** Marker−trait associations identified for agro-morphological traits of potato using MLM approaches in the software program TASSEL.

Traits	Locus	Chr.	P value	Q-value	PVE (%)	Estimate
Starch Content	STI032_4	5	9.24E-06	1.21E-03	12.56	-1.77
	STI032_6	5	2.27E-04	1.49E-02	8.86	1.56
	SSR08337_1	4	8.54E-04	3.73E-02	7.31	1.35
Growth Period	STI032_4	5	3.56E-05	4.67E-03	11.01	-13.18
	STI0012-3	4	1.08E-04	7.07E-03	9.72	-17.14

P value, the significance level of marker−trait associations using TASSEL software; Q-value, the significance level of false discoery rate (FDR) analysis in R software; PVE(%), phenotypic variation explained; Estimate, the value of gene effect estimated using TASSEL software.

## Discussion

### Evaluation of cultivated potato germplasm resources

Generally, phenotypic traits are considered to be the basic elements for selecting clones from cross-combinations during potato breeding. The traits of plant maturity, tuber starch content, reducing sugar content, tuber shape, skin color and flesh color are widely used to select varieties in potato improvement programs (Jansky et al., 2009; Xu and Jin, 2017). Twelve phenotypic traits were evaluated in the study to stress the interaction between phenotypic variability and genetic diversity (**Table 1**, **Figure 1**).

Cluster analysis has been widely used for classifying plant genetic material. Dendrograms of the cluster analysis based on morphological and physiological traits for rice (Verma et al., 2019), tomato (Ayenan et al., 2021) and sweet potato (Meng et al., 2021). In this study, most traits of 149 cultivars showed significant differences. The dendrogram exhibited three large cultivar groups with significantly different values for several traits (**Supplementary Figure S1**). Our findings provide useful information for selecting parents for hybrid combinations and identifying similar varieties for market supervision.

SSR markers have been widely used to estimate genetic diversity in many potato populations. The results of our study indicated that the average number of alleles per marker was 5.46 and the mean value of *PIC* was 0.702, demonstrating a high level of heterozygosity in tetraploid cultivated potato accessions. These results were consistent with those reported in previous studies (Kishine et al., 2017; Wang et al., 2019) for markers 31924, 43016, STG0025 and STM0037. However, lower allele numbers for markers S118, S170, S182, S187, S189, S192 and S7 were observed compared with those reported by Duan et al. (2019). Duan et al. (2021) reported a gene diversity of 0.258 in 189 potato genotypes, including 61 wild *Solanum* species genotypes, 32 *S. tuberosum Andienum* genotypes, 87 *S. tuberosum Chilotanum* genotypes, and 9 complex *Solanum* hybrids of diploids. Wang et al. (2019) reported a gene diversity of 0.309 in 292 potato germplasms of foreign elite lines, local landraces and cultivars in China. Research on the Colombian Central Collection of *S. tuberosum group Andigenum* showed a genetic diversity of 0.252-0.319 (Berdugo-Cely et al., 2017). A total of 214 advanced potato clones analyzed by the Infinium 22 K Potato Array revealed an overall average gene diversity of 0.59 (Pandey et al., 2021). Moreover, genotyping of 288 potato (*Solanum tuberosum L*.) accessions using SSR and AFLP markers revealed a genetic diversity of 0.311-0.367 (Wang et al., 2017), which is lower than that observed in the present study. The differences in the number of alleles might be due to the high genetic diversity of materials and highly polymorphic markers in the study.

Potato is a tetraploid outcrossing crop, and its heterozygosity is usually higher than expected (Machida-Hirano, 2015; Meirmans et al., 2018). In the present study, we detected a high level of genetic diversity in the population, and the expected and observed heterozygosity varied from 0.392 to 0.811 and 0.460 to 0.988, respectively. The mean value of observed heterozygosity (*Ho*) was higher than that of expected heterozygosity (*He*) in the study (**Table 2**). Additionally, the *PIC* varied from 0.462 to 0.813 with an average of 0.702, and the population *F_IS_* changed from − 0.359 to 0.551 with an average of -0.025, of which 15 SSRs were negative, showing that there was significant heterozygous redundancy in the experimental potato varieties. This may be related to self-incompatibility introgression and heterozygosity of cultivated potato germplasm (Xu and Jin, 2017), which agrees with previously reported results (Meirmans et al., 2018). In addition, mutation positive selection and heterosis in the evolution process are also important reasons for the high heterozygosity of potato (Machida-Hirano, 2015).

### Genetic relationship among the population

The detection of genetic structure is an important part of population genetic studies. Multiple factors, such as population size, natural selection, genetic drift and evolutionary history, can affect the genetic structure of potato germplasm. Wang et al. (2019) reported that based on collection sites, two main groups were subdivided into seven groups through Structure analysis. Wang et al. (2017) reported that global potato accessions could be classified into seven subgroups and an admixture group through Bayesian analysis. Pandey et al. (2021) also reported three groups of advanced clones based on potato marker classes detected by a potato breeding program in the USA. 189 potato genotypes were divided into five subgroups by STRUCTURE analysis (Duan et al., 2021). In this study, we compared the results of Structure analysis (**Figure 3D**) and phylogenetic tree clustering (**Figure 4**). Clustal I contains 8 varieties from the Q1 group, accounting for 88.9% of all Q1 varieties; Clustal II contains 63 varieties from the Q2 group, accounting for 85.1% of all Q2 varieties; and Clustal III contains 42 varieties from the Q3 group, accounting for 63.7% of Q3 varieties (**Figure 4**). If the similarity ancestry threshold is set at 60% (Zhao et al., 2010), 7 cultivars belong to the Q1 group, and Zhongshu 33 and Mengshu 12 are admixtures. Forty-eight cultivars belong to the Q2 group, 45 cultivars belong to the Q3 group, and the rest are mixtures. This result is consistent with the phylogenetic tree clustering. The difference between Structure analysis and phylogenetic tree clustering may be due to the different algorithms. The cluster results of Structure analysis (**Figure 3D**) and PCoA in this study were similar to those of previous reports (Duan et al., 2019; Duan et al., 2021), except for ‘Longshu 7’, ‘Yanshu 4’ and ‘Fujin’. The grouping patterns or numbers were different from those in other studies, which may be caused by the use of different markers and populations.

The results of word cloud and network analysis indicated that seven potato varieties (cvs. Schwalbe, Zhongshu 3, Epoka, C93.154, Katahdin, Duozibai and Shepody) were highly frequently parental in these potato accessions (**Figure 2**). Similar results have been reported previously, demonstrating that the parents of Schwalbe, Katahdin, Epoka, Zhongshu 3, Duozibai and Shepody are often used extensively (Li et al., 2018a; Lee et al., 2021). Combined with the Structure data, Zhongshu 9 (No. 144) is the offspring of the cross combination of Zhongshu 3 (No. 139) × Shepody (No. 96), and Zhongshu 33 (No. 124) is the progeny of the cross combination of Zhongshu 3 (No. 139) × Zaodabai (No. 118). The introgression of the hybrids can be confirmed in the study (**Figure 3D**). In addition, five out of seven offspring of Schwalbe, five out of six offspring of Epoka, and three offspring of Mira were all in the second green group (**Figure 3D**). Four of the five descendants of C93.154 and the three descendants of Desiree belong to the third blue group (**Figure 3D**). The AMOVA results indicated 90% variation within the groups and 10% variation among groups, which is similar to previous results (Wang et al., 2019; Duan et al., 2021).

### Markers associated with starch content and growth period traits

Many studies have focused on QTLs for maturity and starch content in different populations in recent years. Li et al. (2019) identified eleven QTLs for tuber starch content from seven chromosomes and six QTLs for plant maturity from five chromosomes. A QTL was detected at 84 cM on chromosome 5, contributing to 33.55% of the variation in plant maturity (Li et al., 2018b). A major QTL on chromosome V for plant maturity was identified (Bradshaw et al., 2008). Researchers cloned the gene *StCDF1*, which encodes a DOF transcription factor that regulates tuberization and plant life cycle length (Kloosterman et al., 2013). A candidate SNP marker for plant maturity near the *StCDF1* gene was identified through SNP genetic mapping (Massa et al., 2015). In this study, we detected 4 SSRs located on chromosomes IV and V that showed significant genetic effects on maturity and starch content traits based on a false discovery rate (FDR) method test (**Table 3**), which enhanced the reliability of the association results.

As in previous reports, we also found a significant correlation between growth period and tuber starch content traits in potato (**Figure 1**) (Schönhals et al., 2016; Li et al., 2018b; Li et al., 2019). The starch content in late-maturing genotypes was higher than that in early-maturing cultivars, probably because more time was available to accumulate starch. Interestingly, the allele tagged by STI032_4 was negatively associated with both growth period and starch content. In contrast, the allele tagged by STI032_6 was positively associated with starch content traits. In contrast, the marker STI032 was only associated with plant maturity in 192 genotypes from the Longshu 8 × Zaodabai cross in a previous report (Li et al., 2019).

In conclusion, we analyzed the genetic diversity of 149 cultivated potato varieties in China using phenotypic traits and molecular SSR markers, and alleles of the STI032 marker with a significant correlation with starch content and growth period were detected. The study provided a useful tool for genetic analysis, varietal identification, breeding improvement and more extensive studies in potato.

## Data availability statement

The original contributions presented in the study are included in the article/**Supplementary Materials**. Further inquiries can be directed to the corresponding author.

## Author contributions

LJ designed the experiment; JH and MM performed the SSR analysis, MM, JX, SD, and CB provided the samples; JH, MM, and FJ analyzed the data and prepared the manuscript, LJ, GL, XW, and JH edited the manuscript. All authors read and confirmed the manuscript.

## Funding

This research was funded by Agricultural Breeding Project of Ningxia Hui Autonomous Region, China, grant number 2019NYYZ01-1, Science and Technology Project of Inner Mongolia Autonomous Region, China, grant number 2021ZD0005, China Agriculture Research System, grant number CARS-9.

## Conflict of interest

The authors declare that the research was conducted in the absence of any commercial or financial relationships that could be construed as a potential conflict of interest.

## Publisher’s note

All claims expressed in this article are solely those of the authors and do not necessarily represent those of their affiliated organizations, or those of the publisher, the editors and the reviewers. Any product that may be evaluated in this article, or claim that may be made by its manufacturer, is not guaranteed or endorsed by the publisher.
