# Supplementary material for: Phenotypic variability and genetic diversity analysis of cultivated potatoes in China
Source: Front Plant Sci. 2022 Sep 23;13:954162. doi: 10.3389/fpls.2022.954162 (PMC9541749; doi:10.3389/fpls.2022.954162)
Supplement: Supplementary file 1 [file DataSheet_1.docx]

Supplementary Material

# Supplementary Figures and Tables


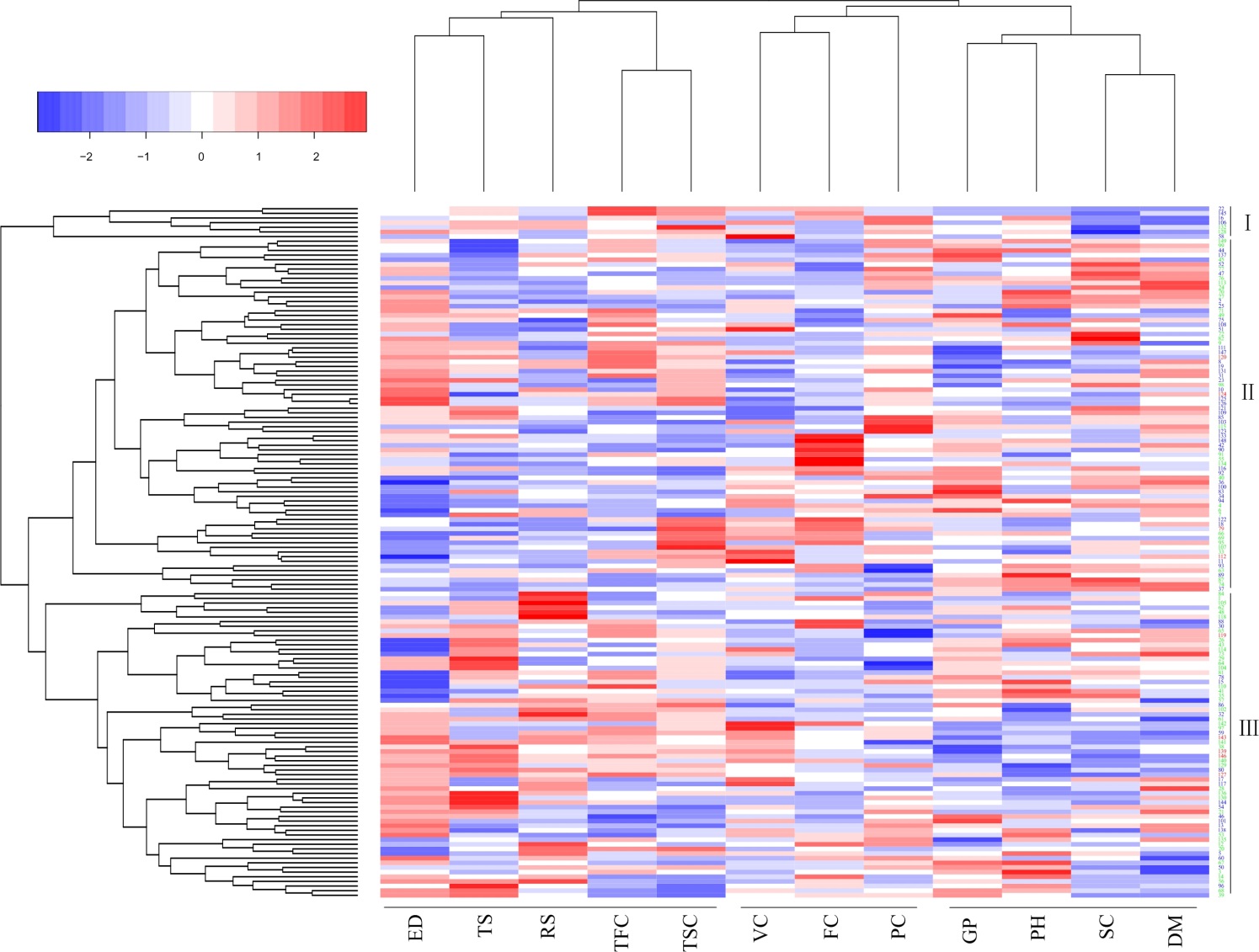
­

**Supplementary Figure S1.** Dendrogram showing cluster patterns of 12 plant and tuber traits in 149 potato varieties. RS: reducing sugar content; TS: tuber shape; ED: eye depth; TFC: tuber flesh color; TSC: tuber skin color; VC: vitamin C content; FC: flower color; PC: protein content; SC: starch content; GP: growth period; DM: dry matter content; PH: plant height. The population was divided into three groups from top to bottom.


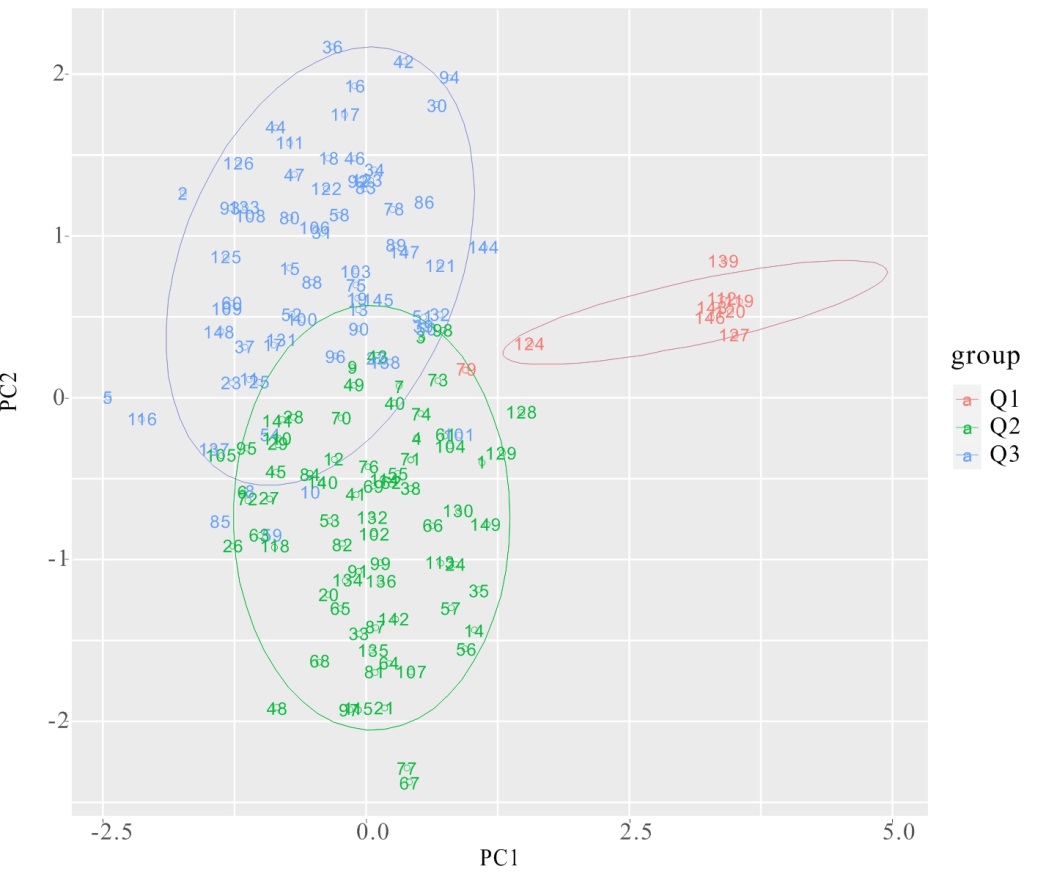


**Supplementary Figure S2.** Principal coordinate analysis of 149 potato cultivars using SSR markers. Note: Numbering of genotypes corresponds to the code numbers in Supplementary Table S1.


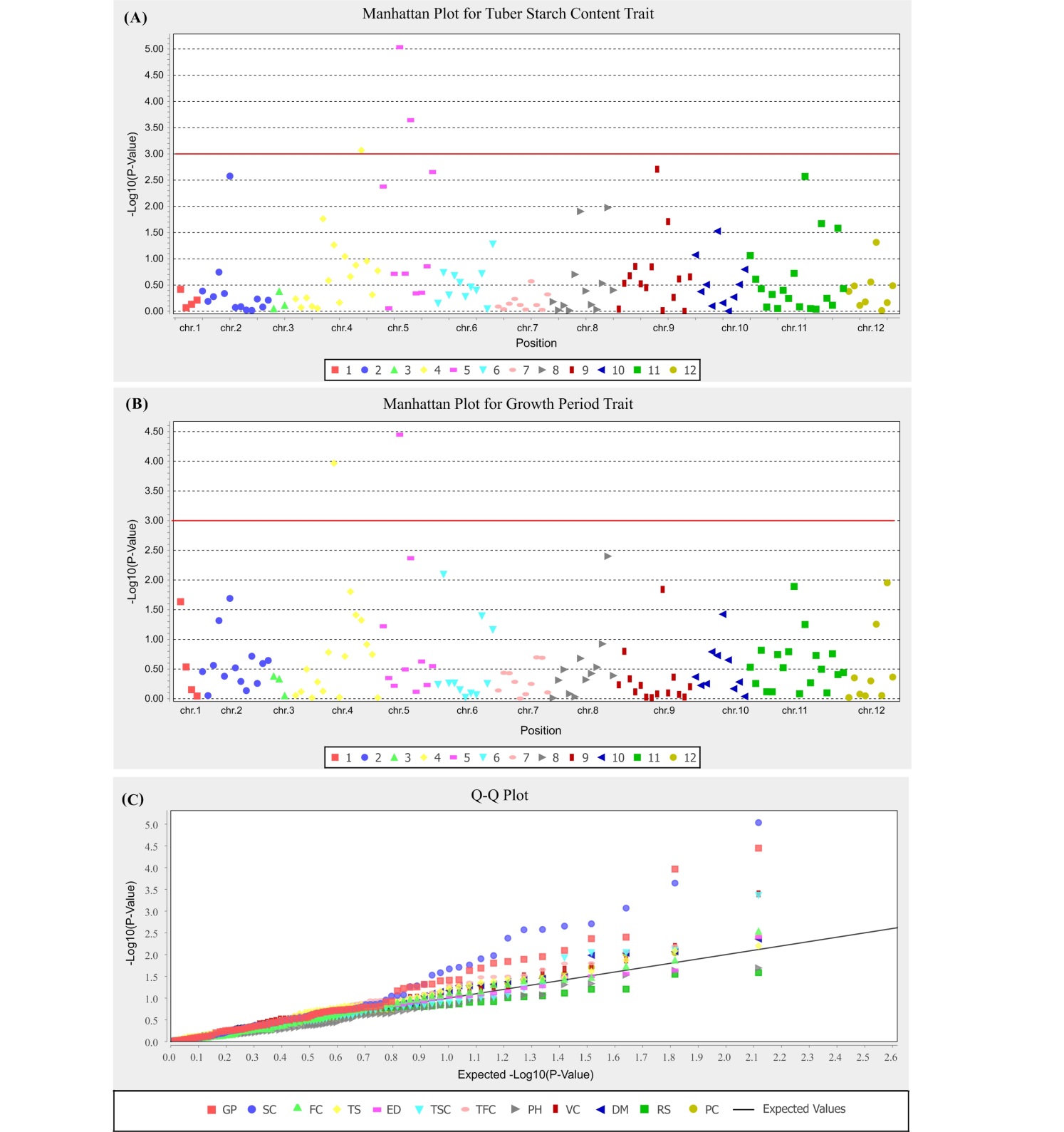


**Supplementary Figure S3.** Manhattan plot showing significant marker-trait associations (MTAs) identified for tuber starch content (A) and growth period (B) traits in the potato population using a mixed linear model (MLM) in TASSEL program. The significant MTAs for two traits are detected above threshold. QQ plots obtained during study of marker–trait associations for 12 traits in the potato population (C). The figure shows QQ plots for all the 12 traits. GP: growth period; SC: starch content; FC: flower color; TS: tuber shape; ED: eye depth; TSC: tuber skin color; TFC: tuber flesh color; PH: plant height; VC: vitamin C content; DM: dry matter content; RS: reducing sugar content; PC: protein content.

**Supplementary Table** S**1**. List of cultivars used in the present study

| Code | Cultivar | Parentage | Breeding program |
| --- | --- | --- | --- |
| 1 | Anshu 56 | 175×Kexin 2 | Ankang Institute of Agricultural Sciences, Shaanxi Provinces |
| 2 | Atlantic | B5141-6×Wauseon | Imported from United States of American |
| 3 | Bashu 10 | Hutou×Schwalbe | Zhangjiakou Bashang Institute of Agricultural Sciences , Hebei Province |
| 4 | Bashu 7 | 35-131×73-21-1 | Zhangjiakou Bashang Institute of Agricultural Sciences , Hebei Province |
| 5 | Bashu 9 | Duozibai×Epoka | Zhangjiakou Bashang Institute of Agricultural Sciences , Hebei Province |
| 6 | Changshu 4 | Self inbred progeny of Epoka | Potato Research Institute, Heilongjiang Academy of Agricultural Sciences |
| 7 | Chaobai | 372－18×Kexin 3 | Dalian Institute of Agricultural Sciences, Liaoning Province |
| 8 | Chuanyu 39 | 379645.4×7XY-1 | Crop Research Institute, Sichuan Academy of Agricultural Sciences |
| 9 | Chuanyu 10 | 44-4×Liangshu 3 | Crop Research Institute, Sichuan Academy of Agricultural Sciences |
| 10 | Chuanyu 4 | C1an-dia×7XY-1 | Crop Research Institute, Sichuan Academy of Agricultural Sciences |
| 11 | Chuanyu 6 | 44-4×Liangshu 3 | Crop Research Institute, Sichuan Academy of Agricultural Sciences |
| 12 | Chunshu 1 | Red Warba×Katahdin | Institute of Vegetables and Flowers, Jilin Province |
| 13 | Chunshu 5 | Chunshu 2×Ke S2-14-1-12-3-(9) | Institute of Vegetables and Flowers, Jilin Province |
| 14 | Cooperation 001 | True seed from CIP | Huize Agricultural Technology Center and the Root and Tuber Crops Institute of Yunnan Normal University |
| 15 | Cooperation 23 | True seed from CIP | Huize Agricultural Technology Center and the Root and Tuber Crops Institute of Yunnan Normal University |
| 16 | Cooperation 88 | “I-1085”×BLK2 | Huize Agricultural Technology Center and the Root and Tuber Crops Institute of Yunnan Normal University |
| 17 | Deshu 2 | Hui-2×PB06 | Dehong Institute of Agricultural Sciences; Industrial Crops Research Institute, Yunnan Academy of Agricultural Sciences |
| 18 | Desiree | Uurgenta×Depesc | Imported from Netherlands |
| 19 | Dongnong 303 | Anemome×Katahdin | Northeast Agricultural University |
| 20 | Dongnong 304 | S4-5-3-9-1-25-(6)×NS12-156-(1) | Northeast Agricultural University |
| 21 | Dongnong 305 | Atlantic×NS12-156-1-1 | Northeast Agricultural University |
| 22 | Dongnong 306 | All Blue | Northeast Agricultural University |
| 23 | Dongnong 307 | DDGS-1 | Northeast Agricultural University |
| 24 | Dongnong 308 | W4×Ns79-12-1 | Northeast Agricultural University |
| 25 | Emalingshu 1 | 674－5×c.f.k－69.1 | Enshi Southern China Potato Research Center, Hubei Province |
| 26 | Emalingshu 10 | wensheng11×dorita518 | Enshi Southern China Potato Research Center, Hubei Province |
| 27 | Emalingshu 14 | T962-25×IX-38-6 | Enshi Southern China Potato Research Center, Hubei Province |
| 28 | Emalingshu 3 | 7914-33×59-5-86 | Enshi Southern China Potato Research Center, Hubei Province |
| 29 | Emalingshu 5 | 393143-12×NS51-5 | Enshi Southern China Potato Research Center, Hubei Province |
| 30 | Favorita | ZPC50-35×ZPC55-37 | Imported from Netherlands |
| 31 | Fujin | 8837-2×Youjin | Benxi Institute of Agricultural Sciences, Liaoning Province |
| 32 | Fuke 212 | Yanzi×KeXin2 | Institute of Crop, Fujian Academy of Agricultural Sciences |
| 33 | FuKe 76 | Bashu9×Katahdin | Institute of Crop, Fujian Academy of Agricultural Sciences;  Longyan Institute of Agricultural Sciences, Gansu Province |
| 34 | Gaoyuan 1 | Niutou×Duozibai | Qinghai Academy of Agricultural and Forestry Sciences |
| 35 | Gaoyuan 4 | Duozibai×Mira | Qinghai Academy of Agricultural and Forestry Sciences |
| 36 | Gaoyuan 5 | Shenyanwo×742 | Qinghai Academy of Agricultural and Forestry Sciences |
| 37 | Gaoyuan 7 | Gaoyuan 4×Gaoyuan 3 | Qinghai Academy of Agricultural and Forestry Sciences |
| 38 | Huangmazi | Unknown | Local Cultivar in Wangkui County, Heilongjiang Province |
| 39 | Hui2 | Yinxike×Weihui2 | Huize Agricultural Technology Center, Yunnan Province |
| 40 | Hutou | Zishanyao×B76-16 (Xiaoyezi) | Zhangjiakou Bashang Institute of Agricultural Sciences , Hebei Province |
| 41 | Jinshu 11 | H319－1×NT/TBULK | High Latitude Crops Institute, Shanxi Academy of Agricultural Sciences |
| 42 | Jinshu 12 | 75-30-7×Schwalbe | Wuzhai Experimental Station, Shanxi Academy of Agricultural Sciences |
| 43 | Jinshu 14 | 9201-59×(6401-3-35×Schwalbe) | High Latitude Crops Institute, Shanxi Academy of Agricultural Sciences |
| 44 | Jinshu 5 | Jinshu 2×Schwalbe | High Latitude Crops Institute, Shanxi Academy of Agricultural Sciences |
| 45 | Jinshu 7 | 6401-3-35×Schwalbe | High Latitude Crops Institute, Shanxi Academy of Agricultural Sciences |
| 46 | Jizhangshu 8 | 720087×X4.4 | High Latitude Crops Institute, Hebei Provinces |
| 47 | Kexin 22 | AMYLEX×8y-220/1 | Keshan branch of Heilongjiang Academy of Agricultural Sciences |
| 48 | Kexin 1 | 374-128×Epoka | Potato Research Institute, Heilongjiang Academy of Agricultural Sciences |
| 49 | Kexin 11 | CIP7176×Epoka | Potato Research Institute, Heilongjiang Academy of Agricultural Sciences |
| 50 | Kexin 13 | Two generation self inbred progeny of Mira | Potato Research Institute, Heilongjiang Academy of Agricultural Sciences |
| 51 | Kexin 14 | S16-1-1-14-1-3-6-(5)×A-11-1-8-(9) | Potato Research Institute, Heilongjiang Academy of Agricultural Sciences |
| 52 | Kexin 15 | Belmont×Hu 8342-36 | Potato Research Institute, Heilongjiang Academy of Agricultural Sciences |
| 53 | Kexin 16 | Beifanghong×KeBP9601 | Potato Research Institute, Heilongjiang Academy of Agricultural Sciences |
| 54 | Kexin 17 | F81109×B5141-6 | Potato Research Institute, Heilongjiang Academy of Agricultural Sciences |
| 55 | Kexin 18 | Epoka×374-128 | Potato Research Institute, Heilongjiang Academy of Agricultural Sciences |
| 56 | Kexin 19 | Kexin 2×KPS92-1 | Potato Research Institute, Heilongjiang Academy of Agricultural Sciences |
| 57 | Kexin 2 | Mira×Epoka | Potato Research Institute, Heilongjiang Academy of Agricultural Sciences |
| 58 | Kexin 4 | Anemome×Katahdin | Potato Research Institute, Heilongjiang Academy of Agricultural Sciences |
| 59 | Kexin 5 | Anemome×Katahdin | Potato Research Institute, Heilongjiang Academy of Agricultural Sciences |
| 60 | Kexin 6 | S41956×96-56 | Potato Research Institute, Heilongjiang Academy of Agricultural Sciences |
| 61 | Liangshu 97 | 6-36×Schwalbe | Xichang Institute of Agricultural Sciences, Sichuan Province |
| 62 | Liangshu 14 | Self inbred progeny of Kuannae | Xichang Institute of Agricultural Sciences, Sichuan Province |
| 63 | Liangshu 17 | 105-16×Schwalbe | Xichang Institute of Agricultural Sciences, Sichuan Province |
| 64 | Liangshu 3 | Mira×9-49 | Xichang Institute of Agricultural Sciences, Sichuan Province |
| 65 | Liangshu 8 | Liangshu 97×A17 | Xichang Institute of Agricultural Sciences, Sichuan Province |
| 66 | Lishu 1 | Self inbred progeny of Kuannae | Lijiang Institute of Agricultural Sciences, Yunnan Province |
| 67 | Lishu 2 | Huzi 79-172×NS79-12-1 | Lijiang Institute of Agricultural Sciences, Yunnan Province |
| 68 | Lishu 6 | A10—39×NS40—37 | Lijiang Institute of Agricultural Sciences, Yunnan Province |
| 69 | Lishu 7 | Kendor×ALAMO | Lijiang Institute of Agricultural Sciences, Yunnan Province |
| 70 | Longshu 1 | Cornelia×Changshu 4 | Institute of Potato, Gansu Academy of Agricultural Sciences |
| 71 | Longshu 10 | Zhuangshu3×Fitrolay | Institute of Potato, Gansu Academy of Agricultural Sciences |
| 72 | Longshu 3 | 35-131×73-21-1 | Institute of potato, Gansu Academy of Agricultural Sciences |
| 73 | Longshu 4 | 62-47/119-ll | Institute of potato, Gansu Academy of Agricultural Sciences |
| 74 | Longshu 6 | Wushu 86-6-14×Longshu 4 | Institute of Potato, Gansu Academy of Agricultural Sciences |
| 75 | Longshu 7 | Zhuangshu3×Fitrolay | Institute of Potato, Gansu Academy of Agricultural Sciences |
| 76 | Longshu 9 | 93-10-237×G-13-1 | Institute of Potato, Gansu Academy of Agricultural Sciences |
| 77 | Mengshu 10 | Hudan 81-118×Hudan 80-298 | Hulun Buir Institute of Agricultural Sciences, Inner Mongolia Autonomous Region |
| 78 | Mengshu 11 | Unknown | Ulanqab Institute of Agricultural Sciences, Inner Mongolia Autonomous Region |
| 79 | Mengshu 12 | 546×Hudan 81-149 | Hulun Buir Institute of Agricultural Sciences, Inner Mongolia Autonomous Region |
| 80 | Minshu 1 | Favorita×Atlantic | Longyan Institute of Agricultural Sciences, Gansu Province |
| 81 | Mira | Capella×B.R.A.089 | Imported from Germany |
| 82 | Neishu 7 | Hudan 80-298×Hu 8206 | Hulun Buir Institute of Agricultural Sciences, Inner Mongolia Autonomous Region |
| 83 | Ningshu 4 | Self inbred progeny of Lanhuayangyu | Guyuan Institute of Agricultural Sciences, Ningxia Hui Autonomous Region |
| 84 | Ningshu 5 | Self inbred progeny of 76-2-15 | Guyuan Institute of Agricultural Sciences, Ningxia Hui Autonomous Region |
| 85 | Ningshu 7 | Ningshu1×(Aputa×71-18-2) | Guyuan Institute of Agricultural Sciences, Ningxia Hui Autonomous Region |
| 86 | Qingshu 168 | Fushen 6-3×Desiree | Qinghai Academy of Agricultural and Forestry Sciences |
| 87 | Qingshu 2 | Gaoyuan 4×magura | Qinghai Academy of Agricultural and Forestry Sciences |
| 88 | Qingshu 3 | Shenyanwo×Gaoyuan 3 | Qinghai Academy of Agricultural and Forestry Sciences |
| 89 | Qingshu 4 | Niutou×Desiree | Qinghai Academy of Agricultural and Forestry Sciences |
| 90 | Qingshu 6 | Gu33-1×92-9-44 | Qinghai Academy of Agricultural and Forestry Sciences |
| 91 | Qingshu 7 | 92-32-42×92-5-2 | Qinghai Academy of Agricultural and Forestry Sciences |
| 92 | Qingshu 8 | Qingshu 2×Tuodu 175 | Qinghai Academy of Agricultural and Forestry Sciences |
| 93 | Qingshu 9 | 387521.3×APHRODITE | Institute of Crop Research, Qinghai Academy of Agricultural and Forestry Sciences |
| 94 | Qinyu 31 | Yun 94-51×89-1 | Ankang Institute of Agricultural Sciences, Shaanxi Provinces |
| 95 | Shengli 1 | 63-8-27×62-1-10 | Institute of Potato, Gansu Academy of Agricultural Sciences |
| 96 | Shepody | Shepody | Imported from Canada |
| 97 | Shuangfeng 5 | IROSE×Fengshoubai | Institute of Vegetables, Shandong Academy of Agricultural Sciences |
| 98 | Shuangfeng 6 | 83119-(10)×PVY-31 | Institute of Vegetables, Shandong Academy of Agricultural Sciences |
| 99 | Tianshu 10 | Zhuangshu3×Zhenshu1 | Tianshui Institute of Agricultural Sciences, Gansu Province |
| 100 | Tianshu 8 | 62-118×DTO-33 | Tianshui Institute of Agricultural Sciences, Gansu Province |
| 101 | Tianshu 9 | 91-26-116×85-6-14 | Tianshui Institute of Agricultural Sciences, Gansu Province |
| 102 | Tongshu 9 | Liwaihaung×Argo | High Latitude Crops Institute, Shanxi Academy of Agricultural Sciences |
| 103 | Weishu 8 | Unknown | Huichuan Farm inWeiyuan County, Gansu Province |
| 104 | Weiyu 3 | Self inbred progeny of Kuannae | Weining Institute of Agricultural Sciences, Guizhou Province |
| 105 | Xiaoyezi | B76-16(96-44×528-170) | Former Central Agricultural Institute |
| 106 | Xingjia 2 | gloria×21-36-27-31 | Heilongjiang greater hinggan mountains region agriculture forestry research institute |
| 107 | Xishu 1 | Self inbred progeny of Duozibai | High Latitude Crops Institute, Shanxi Academy of Agricultural Sciences |
| 108 | Xuanshu 2 | ECSort×CFK69.1 | Xuanwei Agricultural Technology Center, Yunnan Province |
| 109 | Xuanshu 5 | Vytok × 387136.14 | Yunnan Xuanwei potato seed potato research and Development Center;  Industrial Crops Research Institute, Yunnan Academy of Agricultural Sciences |
| 110 | Yanshu 4 | Liesiji from Moscow | Yanbian Korean Autonomous Prefecture Academy of Agricultural Sciences, Jilin Province |
| 111 | Youjin | NS80-31×8023-10 | Benxi Institute of Agricultural Sciences, Liaoning Province |
| 112 | Yunshu 101 | S95-105×Neishu 7 | Industrial Crops Research Institute, Yunnan Academy of Agricultural Sciences |
| 113 | Yunshu 102 | S95-105×Neishu 7 | Industrial Crops Research Institute, Yunnan Academy of Agricultural Sciences |
| 114 | Yunshu 201 | S95-105×Neishu 7 | Industrial Crops Research Institute, Yunnan Academy of Agricultural Sciences |
| 115 | Yunshu 301 | 93-92×C89-94 | Industrial Crops Research Institute, Yunnan Academy of Agricultural Sciences |
| 116 | Yunshu 401 | Line3258×White Flower Atlantic | Industrial Crops Research Institute, Yunnan Academy of Agricultural Sciences;  Institude of Zhaotong Agricultural Science and Technology; Huize Agricultural Technology Center |
| 117 | Yunshu 505 | Serrana×YAKHANT | Industrial Crops Research Institute, Yunnan Academy of Agricultural Sciences;  Dehong Institute of Agricultural Sciences |
| 118 | Zaodabai | Wulibai×74-128 | Benxi Institute of Agricultural Sciences, Liaoning Province |
| 119 | Zhengshu 6 | Gaoyuan 7×Zheng762-93 | Zhengzhou Institute of Vegetables, Henan Province |
| 120 | Zhengshu 5 | Gaoyuan 7×Zheng762-93 | Zhengzhou Institute of Vegetables, Henan Province |
| 121 | Zhongda 1 | w2/D-6-1 | Institute of Vegetables and Flowers of Chinese Academy of Agricultural Sciences.  Daxinganling Institute of Agricultural Sciences |
| 122 | Zhongshu 26 | C92.140×C93.154 | Institute of Vegetables and Flowers, Chinese Academy of Agricultural Sciences |
| 123 | Zhongshu 27 | LR93.309×C93.154 | Institute of Vegetables and Flowers, Chinese Academy of Agricultural Sciences |
| 124 | Zhongshu 33 | Zaodabai×Zhongshu3 | Institute of Vegetables and Flowers, Chinese Academy of Agricultural Sciences |
| 125 | Zhongshu 10 | F79055×ND860-2 | Institute of Vegetables and Flowers of Chinese Academy of Agricultural Sciences.  Potato Research Centre, Agriculture and Agri-Food Canada |
| 126 | Zhongshu 11 | Aminca×Chaleur | Institute of Vegetables and Flowers of Chinese Academy of Agricultural Sciences.  Potato Research Centre, Agriculture and Agri-Food Canada |
| 127 | Zhongshu 12 | W953×FL475 | Institute of Vegetables and Flowers, Chinese Academy of Agricultural Sciences |
| 128 | Zhongshu 13 | Shepody×Zhongshu 3 | Institute of Vegetables and Flowers, Chinese Academy of Agricultural Sciences |
| 129 | Zhongshu 14 | Shepody×Zhongshu 3 | Institute of Vegetables and Flowers, Chinese Academy of Agricultural Sciences |
| 130 | Zhongshu 15 | Shepody×Zhongshu3 | Institute of Vegetables and Flowers, Chinese Academy of Agricultural Sciences |
| 131 | Zhongshu 16 | W738×S477 | Institute of Vegetables and Flowers, Chinese Academy of Agricultural Sciences |
| 132 | Zhongshu 17 | 881-19×Zhongshu6 | Institute of Vegetables and Flowers, Chinese Academy of Agricultural Sciences |
| 133 | Zhongshu 18 | C91.628×C93.154 | Institute of Vegetables and Flowers, Chinese Academy of Agricultural Sciences |
| 134 | Zhongshu 19 | 92.187×C93.154 | Institute of Vegetables and Flowers, Chinese Academy of Agricultural Sciences |
| 135 | Zhongshu 2 | LT-2×DTO-33 | Institute of Vegetables and Flowers, Chinese Academy of Agricultural Sciences |
| 136 | Zhongshu 20 | LR93.050×92.187 | Institute of Vegetables and Flowers, Chinese Academy of Agricultural Sciences |
| 137 | Zhongshu 21 | LR93.156×92.187 | Institute of Vegetables and Flowers, Chinese Academy of Agricultural Sciences |
| 138 | Zhongshu 22 | PW88065×C93.154 | Institute of Vegetables and Flowers, Chinese Academy of Agricultural Sciences |
| 139 | Zhongshu 3 | Jingfeng 1×BF67A | Institute of Vegetables and Flowers, Chinese Academy of Agricultural Sciences |
| 140 | Zhongshu 4 | Dongnong 3012×85T-13-8 | Institute of Vegetables and Flowers, Chinese Academy of Agricultural Sciences |
| 141 | Zhongshu 5 | Self inbred progeny of ZhongShu 3 | Institute of Vegetables and Flowers, Chinese Academy of Agricultural Sciences |
| 142 | Zhongshu 7 | Zhongshu 2×Jizhangshu 4 | Institute of Vegetables and Flowers, Chinese Academy of Agricultural Sciences |
| 143 | Zhongshu 8 | W953×FL475 | Institute of Vegetables and Flowers, Chinese Academy of Agricultural Sciences |
| 144 | Zhongshu 9 | Shepody×Zhongshu 3 | Institute of Vegetables and Flowers, Chinese Academy of Agricultural Sciences |
| 145 | Zhongshuhong 1 | Kondor×River John Blue | Institute of Vegetables and Flowers, Chinese Academy of Agricultural Sciences |
| 146 | Zhongshuzao 30 | Favorita×Zhongshu5 | Institute of Vegetables and Flowers, Chinese Academy of Agricultural Sciences |
| 147 | Zhongshuzao 35 | F87031×AT12899-02 | Institute of Vegetables and Flowers, Chinese Academy of Agricultural Sciences;  Institute of Vegetables and Flowers, Jinhua City, Zhejiang Province |
| 148 | Zhongxin 24 | Unknown | Chinese Academy of Agricultural Sciences |
| 149 | Zhuangshu 3 | 87-46-1×85-5-1 | Zhuanglang Agricultural Technology Center, Gansu Province |

**Supplementary Table** S**2**. Summary of 24 SSR markers used in the present study

| Primers | Chr. | Forward（5'-3'） | Reverse（5'-3'） | Motif | Fluorescent Dyes |
| --- | --- | --- | --- | --- | --- |
| 31924^a^ | 8 | CGAAGACACCAAATCGCTCAG | GAAACGCCATTAACATTTTACATCG | (ATAC)n | FAM |
| 43016^a^ | 11 | CAAGCTGCATGAAAGCCATC | TTTGCCTAAAAGTTTGTAGTGTGAGG | (ATCC)n | TAMRA |
| S118^b^ | 12 | AGAGATCGATGTAAAACACGT | GTGGCATTTTGATGGATT | Compound(GT/GC)(GT)8 | ROX |
| S151^b^ | 6 | GCTGCTAAACACTCAAGCAGAA | CAACTACAAGATTCCATCCACAG | (AAG)n | ROX |
| S170^b^ | 7 | CGCAAATCTTCATCCGATTC | TCCGGCGGATAATACTTGTT | (GAA)n | FAM |
| S182^b^ | 6 | GGAAGTCCTCAACTGGCTG | TCAACTATATGCCTACTGCCCAA | (TCTT)n | HEX |
| S187^b^ | 4 | CCGTTGATGGGATTGCACA | TGATATTAACCATGGCAGCAGC | (AAG)n | ROX |
| S189^b^ | 9 | CCTTGTAGAACAGCAGTGGTC | TCCGCCAAGACTGATGCA | (AAG)n | FAM |
| S192^b^ | 3 | ACTTCTGCATCTGGTGAAGC | GGTCTGGATTCCCAGGTTG | (ATA)n | ROX |
| S7^b^ | 2 | GACTGGCTGACCCTGAACTC | GACAAAATTACAGGAACTGCAAA | (CA)n(TC)n | HEX |
| SSR08337^c^ | 4 | CGTTAAGGAGGAGGAGGAAAA | CCAAATAACGTGTTGAGCCC | (TG)n | TAMRA |
| STG0025^c^ | 10 | TGGAATCCGAATTACGCTCT | AGGTTTTACCACTCGGGCTT | (AAAC)5 | HEX |
| STG0026^c^ | 7 | ACTGCCGCAAAAAGTGAAAA | GCCGCTAGGTGGAGTAGATG | (CTCC)n | TAMRA |
| STI0012^c^ | 4 | GAAGCGACTTCCAAAATCAGA | AAAGGGAGGAATAGAAACCAAAA | (ATT)n | TAMRA |
| STI017^c^ | 11 | TATGGAAATTCCGGTGATGG | GACGGTGACAAAGAGGAAGG | (CAT)n(TAG)n | TAMRA |
| STI032^c^ | 5 | TGGGAAGAATCCTGAAATGG | TGCTCTACCAATTAACGGCA | (GCA)n | FAM |
| STM0037^d^ | 11 | AATTTAACTTAGAAGATTAGTCTC | ATTTGGTTGGGTATGATA | (TC)5(AC)6(AA(AC)7(AT)4 | FAM |
| STM1049^d^ | 1 | CTACCAGTTTGTTGATTGTGGTG | AGGGACTTTAATTTGTTGGACG | (ATA)6 | FAM |
| STM1104^d^ | 8 | TGATTCTCTTGCCTACTGTAATCG | CAAAGTGGTGTGAAGCTGTGA | (TCT)5 | HEX |
| STM1106^d^ | 10 | TCCAGCTGATTGGTTAGGTTG | ATGCGAATCTACTCGTCATGG | (ATT)13 | HEX |
| STM2022^d^ | 2 | GCGTCAGCGATTTCAGTACTA | TTCAGTCAACTCCTGTTGCG | (CAA)3(CAA)3 | ROX |
| STM3012^d^ | 9 | CAACTCAAACCAGAAGGCAAA | GAGAAATGGGCACAAAAAACA | (CT)4(CT)8 | TAMRA |
| STM5121^d^ | 12 | CACCGGAATAAGCGGATCT | TCTTCCCTTCCATTTGTCA | (TGT)n | ROX |
| STPoAc58^d^ | 5 | TTGATGAAAGGAATGCAGCTTGTG | ACGTTAAAGAAGTGAGAGTACGAC | (TA)n | HEX |

Note: Primer sequences are from the following references: a represent Kishine M. et al. (2017); b represent Duan Y., et al.(2019); c represent Duan Y., et al. (2021); d represent Wang Y., et al. (2019).

**Supplementary Table** S**3**. Alleles peak size of 24 SSR markers primers

| Primers | Chr. | No. of Peaks | No. of profiles | averaged peak size (bp) | | | | | | | | |
| --- | --- | --- | --- | --- | --- | --- | --- | --- | --- | --- | --- | --- |
| 31924 | 8 | 6 | 34 | 164 | 214 | 219 | 223 | 231 | 251 |  |  |  |
| 43016 | 11 | 7 | 27 | 177 | 193 | 197 | 205 | 209 | 221 | 233 |  |  |
| S118 | 12 | 5 | 19 | 141 | 143 | 149 | 161 | 166 |  |  |  |  |
| S151 | 6 | 6 | 28 | 80 | 83 | 91 | 97 | 103 | 105 |  |  |  |
| S170 | 7 | 6 | 34 | 105 | 111 | 117 | 125 | 128 | 144 |  |  |  |
| S182 | 6 | 5 | 19 | 135 | 139 | 144 | 153 | 157 |  |  |  |  |
| S187 | 4 | 5 | 17 | 214 | 217 | 220 | 226 | 240 |  |  |  |  |
| S189 | 9 | 9 | 39 | 176 | 179 | 182 | 185 | 191 | 194 | 197 | 203 | 206 |
| S192 | 3 | 4 | 8 | 157 | 160 | 163 | 172 |  |  |  |  |  |
| S7 | 2 | 7 | 38 | 120 | 128 | 132 | 134 | 138 | 142 | 152 |  |  |
| SSR08337 | 4 | 4 | 10 | 183 | 187 | 189 | 203 |  |  |  |  |  |
| STG0025 | 10 | 2 | 3 | 198 | 202 |  |  |  |  |  |  |  |
| STG0026 | 7 | 4 | 7 | 276 | 280 | 284 | 288 |  |  |  |  |  |
| STI0012 | 4 | 7 | 34 | 167 | 170 | 173 | 175 | 185 | 188 | 191 |  |  |
| STI017 | 11 | 5 | 14 | 160 | 163 | 166 | 169 | 174 |  |  |  |  |
| STI032 | 5 | 6 | 35 | 107 | 110 | 116 | 119 | 122 | 125 |  |  |  |
| STM0037 | 11 | 6 | 39 | 69 | 71 | 75 | 77 | 82 | 84 |  |  |  |
| STM1049 | 1 | 4 | 8 | 179 | 186 | 189 | 199 |  |  |  |  |  |
| STM1104 | 8 | 6 | 18 | 163 | 165 | 168 | 172 | 175 | 177 |  |  |  |
| STM1106 | 10 | 8 | 29 | 122 | 137 | 140 | 150 | 153 | 156 | 159 | 192 |  |
| STM2022 | 2 | 6 | 13 | 172 | 175 | 181 | 187 | 228 | 243 |  |  |  |
| STM3012 | 9 | 5 | 11 | 166 | 194 | 196 | 200 | 210 |  |  |  |  |
| STM5121 | 12 | 4 | 8 | 283 | 286 | 289 | 292 |  |  |  |  |  |
| STPoAc58 | 5 | 4 | 18 | 228 | 232 | 240 | 246 |  |  |  |  |  |

|  | Growth  Period | Starch  Content | Flower  Color | Plant  Height | Vc Cotent | Dry Matter  Content | Reducing Sugar  Content | Protein  Content | Tuber  Shape | Eye  Depth | Tuber Skin  color |
| --- | --- | --- | --- | --- | --- | --- | --- | --- | --- | --- | --- |
| Starch Content | 0.447^**^ |  |  |  |  |  |  |  |  |  |  |
| Flower Color | 0.128 | 0.032 |  |  |  |  |  |  |  |  |  |
| Plant Height | 0.299^**^ | 0.348^**^ | 0.099 |  |  |  |  |  |  |  |  |
| Vc cotent | -0.099 | -0.026 | 0.191 | -0.028 |  |  |  |  |  |  |  |
| Dry Matter Content | 0.272^**^ | 0.564^**^ | 0.034 | 0.214^**^ | 0.006 |  |  |  |  |  |  |
| Reducing Sugar Content | 0.086 | -0.115 | 0.010 | -0.130 | -0.123 | -0.097 |  |  |  |  |  |
| Protein Content | 0.242^**^ | 0.132 | 0.019 | -0.054 | 0.140 | 0.178^*^ | -0.107 |  |  |  |  |
| Tuber Shape | -0.008 | -0.158 | 0.045 | -0.033 | -0.034 | -0.092 | 0.096 | -0.147 |  |  |  |
| Eye Depth | -0.118 | -0.099 | 0.095 | -0.085 | 0.014 | -0.022 | 0.035 | 0.102 | 0.225^**^ |  |  |
| Tuber Skin color | -0.038 | 0.083 | 0.292^**^ | -0.084 | 0.168^*^ | -0.003 | -0.034 | 0.077 | 0.089 | 0.027 |  |
| Tuber Flesh color | -0.062 | -0.051 | 0.121 | -0.039 | 0.000 | -0.166 | -0.017 | -0.045 | 0.161 | 0.162 | 0.531^**^ |
| **: *P*<0.01；*: *P*<0.05 | | | | | | | | | | | |

**Supplementary Table S4**. The correlation analysis of 12 traits of the 149 potato cultivars.

**Supplementary Table** **S5**. Eigenvalues, contribution rate and loading matrix of principal component of 149 potato genotypes

| Importance of components | Comp.1 | Comp.2 | Comp.3 | Comp.4 | Comp.5 | Comp.6 | Comp.7 | Comp.8 | Comp.9 | Comp.10 | Comp.11 | Comp.12 |
| --- | --- | --- | --- | --- | --- | --- | --- | --- | --- | --- | --- | --- |
| Eigenvalues | 1.504 | 1.334 | 1.140 | 1.062 | 1.010 | 1.003 | 0.899 | 0.873 | 0.816 | 0.700 | 0.619 | 0.564 |
| Proportion of Variance | 0.190 | 0.149 | 0.109 | 0.095 | 0.086 | 0.084 | 0.068 | 0.064 | 0.056 | 0.041 | 0.032 | 0.027 |
| Cumulative Proportion | 0.190 | 0.339 | 0.448 | 0.543 | 0.628 | 0.713 | 0.780 | 0.844 | 0.900 | 0.941 | 0.973 | 1.000 |
| Growth Period | 0.437 |  | 0.289 | 0.153 | 0.269 | 0.105 | 0.364 | 0.239 |  | 0.496 |  | 0.405 |
| Starch Content | 0.534 | 0.134 |  |  |  | -0.171 | -0.271 |  |  | 0.285 | -0.396 | -0.579 |
| Flower Color |  | 0.426 |  |  |  | 0.607 |  | -0.392 | 0.467 |  | 0.108 | -0.222 |
| Plant Height | 0.361 |  | 0.233 | -0.301 | -0.362 | 0.216 | 0.354 |  | -0.443 | -0.456 |  |  |
| Vitamin C contentr |  | 0.252 | -0.519 |  |  | 0.466 | -0.223 | 0.359 | -0.452 | 0.219 |  |  |
| Dry Matte contentr | 0.472 |  |  | 0.152 | -0.131 | -0.121 | -0.572 |  | 0.147 | -0.262 | 0.471 | 0.276 |
| Reducing Sugar Content | -0.122 |  | 0.434 | 0.243 | 0.594 | 0.263 | -0.283 |  | -0.405 | -0.219 |  |  |
| Protein Content | 0.21 | 0.154 | -0.368 | 0.553 | 0.219 | -0.193 | 0.391 | 0.154 |  | -0.416 |  | -0.237 |
| Tuber Shape | -0.202 | 0.167 | 0.46 | 0.213 | -0.357 | 0.112 |  | 0.645 | 0.247 | -0.142 |  | -0.165 |
| Eye Depth | -0.159 | 0.209 | 0.127 | 0.604 | -0.428 |  |  | -0.449 | -0.239 | 0.217 | -0.162 | 0.173 |
| Tuber Skin Color |  | 0.602 |  | -0.202 | 0.225 | -0.196 | -0.155 |  |  | -0.207 | -0.494 | 0.428 |
| Tuber Flesh Color | -0.195 | 0.508 | 0.174 | -0.203 |  | -0.389 | 0.137 |  | -0.254 | 0.125 | 0.562 | -0.25 |

**Supplementary Table S6**. Analysis of molecular variance (AMOVA) for the partitioning of microsatellite diversity

| Source of variance | df | SS | MS | Est. Var. | Total variance(%) |
| --- | --- | --- | --- | --- | --- |
| Between Groups | 2 | 210.07 | 105.03 | 2.13 | 10% |
| Within Goups | 146 | 2668.69 | 18.28 | 18.28 | 90% |
| Total | 148 | 2878.75 |  | 20.41 | 100% |
| SS, sum of squares; MS, mean squares. | | | | | |
